# COVID-19-associated rhino-orbital mucormycosis (CAROM)—a case report

**DOI:** 10.1186/s43055-021-00547-5

**Published:** 2021-07-06

**Authors:** Humsheer Singh Sethi, Kamal Kumar Sen, Sudhansu Sekhar Mohanty, Sangram Panda, Kolluru Radha Krishna, Chayasmita Mali

**Affiliations:** 1 Department of Radio-Diagnosis, Kalinga Institute of Medical Sciences, Bhubaneswar, Odisha India; 2 Department of Pathology, Kalinga Institute of Medical Sciences, Bhubaneswar, Odisha India

**Keywords:** COVID-19-associated mucormycosis, Rhino-orbital mucormycosis, COVID-19, Black fungus

## Abstract

**Background:**

There has been a rapid rise in the number of COVID-19-associated rhino-orbital mucormycosis (CAROM) cases especially in South Asian countries, to an extent that it has been considered an epidemic among the COVID-19 patients in India. As of May 13, 2021, 101 CAROM cases have been reported, of which 82 cases were from India and 19 from the rest of the world. On the other hand, pulmonary mucormycosis associated with COVID-19 has a much lesser reported incidence of only 7% of the total COVID-19-associated mucormycosis cases (Singh AK, Singh R, Joshi SR, Misra A, Diab Metab Syndr: Clin Res Rev, 2021). This case report attempts to familiarize the health care professionals and radiologists with the imaging findings that should alarm for follow-up and treatment in the lines of CAROM.

**Case presentation:**

Rhino-orbital mucormycosis (ROM) is a manifestation of mucormycosis that is thought to be acquired by inhalation of fungal spores into the paranasal sinuses. Here, we describe a 55-year-old male, post COVID-19 status with long standing diabetes who received steroids and ventilator therapy for the management of the viral infection. Post discharge from the COVID-19 isolation ICU, the patient complained of grayish discharge from the right nostril and was readmitted to the hospital for the nasal discharge. After thorough radiological and pathological investigation, the patient was diagnosed with CAROM and managed.

**Conclusion:**

Uncontrolled diabetes and imprudent use of steroids are both contributing factors in the increased number of CAROM cases. Our report emphasizes on the radiological aspect of CAROM and reinforces the importance of follow-up imaging in post COVID-19 infection cases with a strong suspicion of opportunistic infections.

## Background

Mucormycosis is an opportunistic angioinvasive disease caused by organisms in the order Mucorales [1]. These organisms are present universally in nature; however, in the background of COVID-19 infection has a high mortality of 30.7% [2]. Prior to the COVID-19 pandemic, the prevalence of mucormycosis in India was approximately 0.14 cases per 1000 population, about 80 times the prevalence in developed countries [3, 4]. With the ongoing second wave of the COVID-19 pandemic, there has been a tremendous increase in the number of ROM cases. Some patients have no option other than debridement surgeries (including enucleation) leaving a substantial percentage of them blind. Here, the role of the radiologist is important to flag such a case and report it early to the treating physician. The most common risk factor in India associated with mucormycosis is diabetes mellitus [5]. To the best of our knowledge, this is the first case report of CAROM from Eastern India.

## Case presentation

**Fig. 1:**
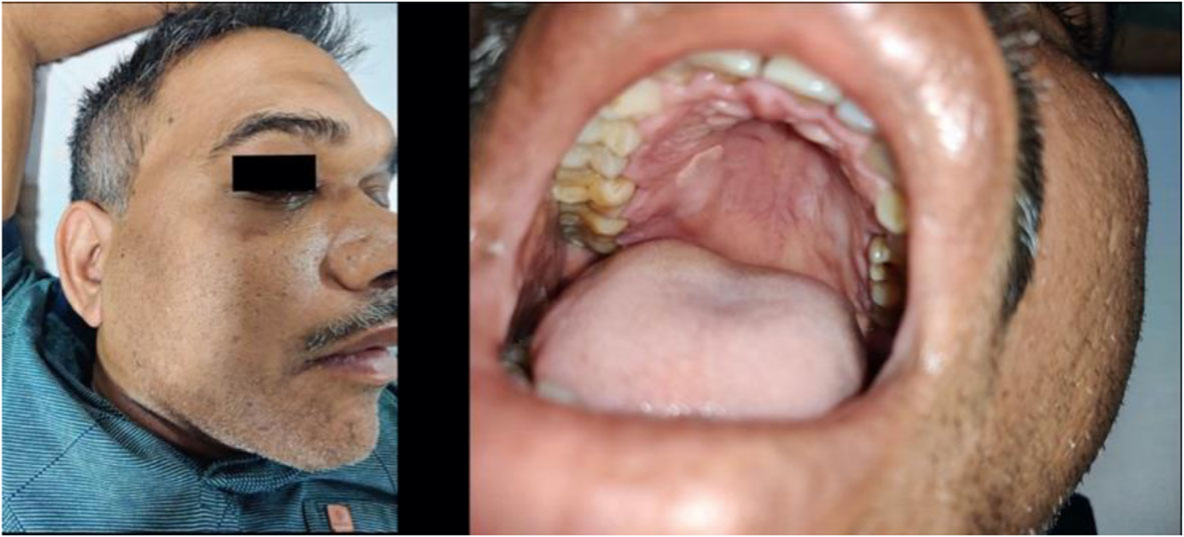
Swelling of the right periorbital area with a plaque-like grayish lesion in the right palate

The 55-year-old male was admitted in the COVID-19 ICU for a period of 2 weeks and treated according to the existing protocols, including steroids and ventilator therapy. On the second day after discharge from the ICU, the patient developed loss of sensation in the skin overlying the malar area with periorbital pain and nasal stuffiness. The next day, he complained of grayish discharge from the right nostril for which he was readmitted in the hospital. The oral examination revealed white crusting over right hard palate (Fig. 1), a finding which was perhaps not appreciated during the first admission. Subsequently, the patient was sent to our department for imaging. A diagnosis of CAROM was established after magnetic resonance imaging (MRI), computed tomography (CT) scan, and microbiological confirmation in a background of COVID-19 infection.

He was a known case of diabetes mellitus type 2 on treatment with human actrapid for 20 years. With HbA1C being 10.7% mg/dl, Inter Leukin-6 being 12 pg/ml, fasting plasma glucose at 182 mg/dl at admission. The coronavirus infection was determined by reverse transcription–polymerase chain reaction (RT-PCR) assays on throat swab samples using a TRUPCR SARS-CoV-2 RT quantitative PCR Kit (in DNA Life Sciences Pvt. Ltd.) and was on non-invasive ventilator support for 8 days.

**Fig. 2:**
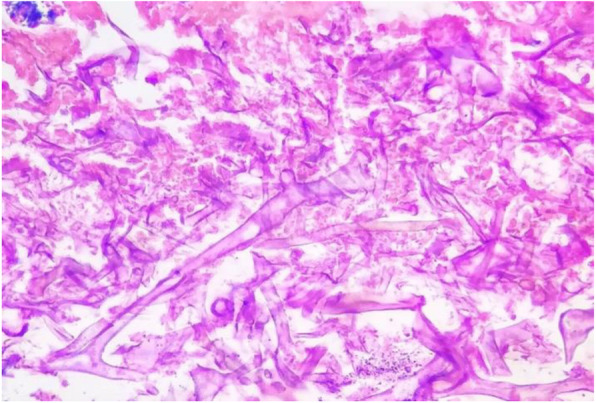
Histopathology smear from right maxillary sinus showing broad nonseptate hyphae of Mucor

Potassium hydroxide (KOH) smear for fungal scraping revealed few fungal spores. Histopathology smear (Fig. 2) from right maxillary sinus showed characteristic broad nonseptate hyphae of Mucor. Wet mount and fungal culture/sensitivity was done from biopsy obtained during debridement. Conservative management was initiated with Amphotericin B and later on with surgical debridement.

## Imaging findings

**Fig. 3:**
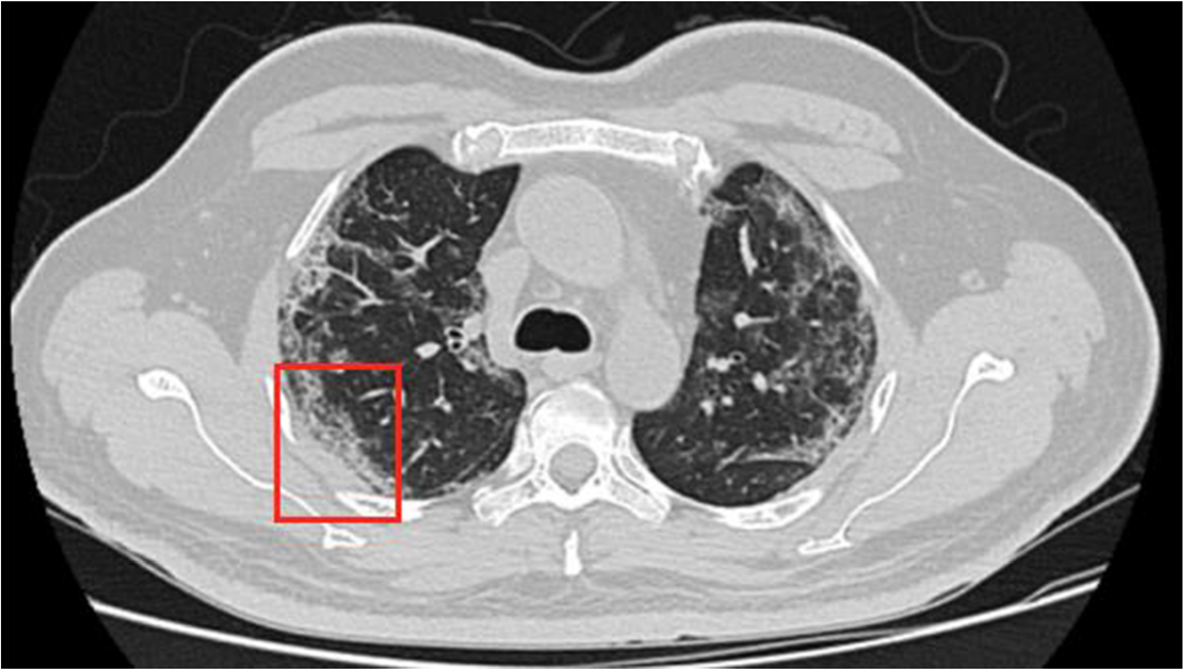
Axial HRCT shows peripherally arranged ground glass opacities with surrounding atelectatic changes in all the lobes of bilateral lungs (red box is one) features typical for COVID-19 with a CT severity score - 17/25

**Fig. 4:**
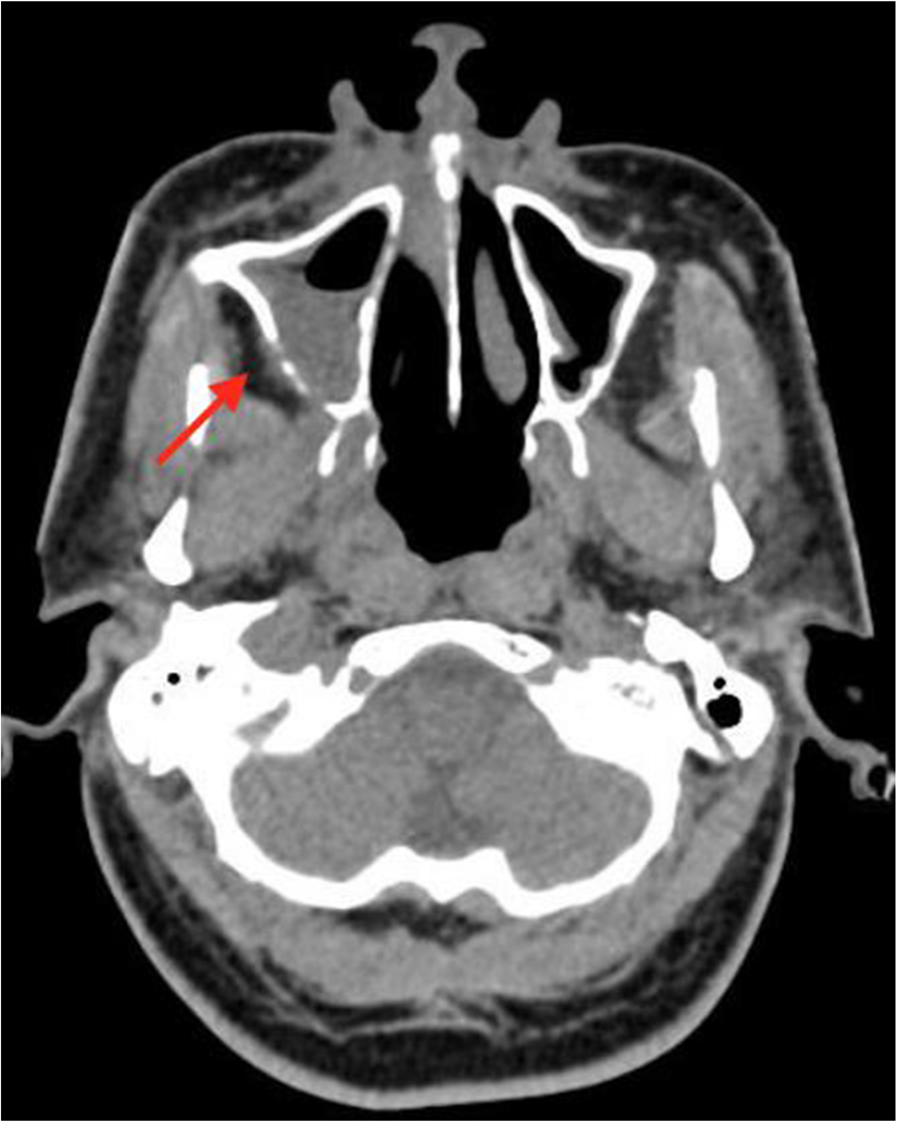
Axial non-contrast CT shows infiltration of the right retroantral fat plane (arrow) indicating invasive disease extending through the posterior wall of the right maxillary sinus

**Fig. 5:**
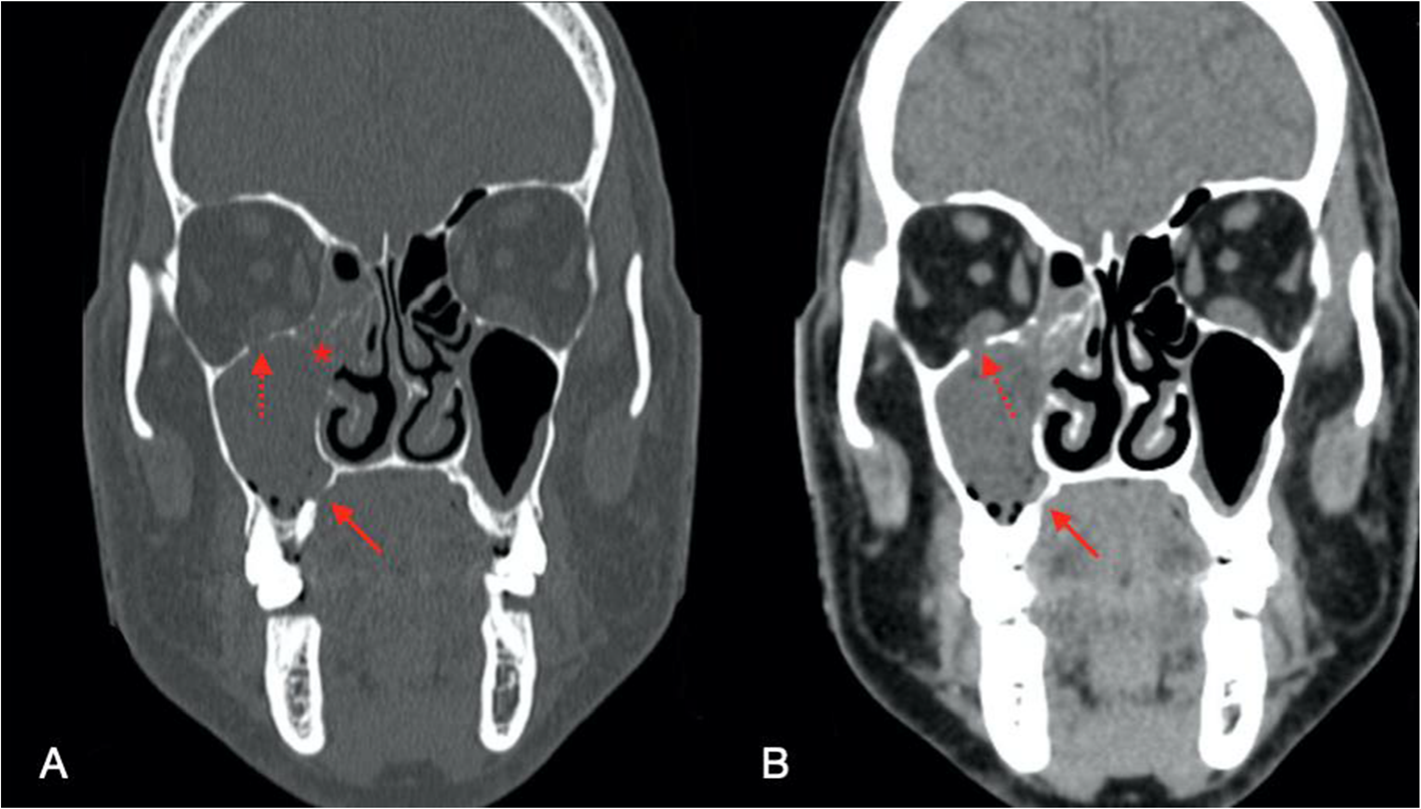
Coronal bone (**A**) and soft tissue (**B**) window demonstrating the mucosal thickening in right ethmoidal and bilateral maxillary sinuses (right > left) with collection in right maxillary sinus. A small defect is noted in postero-lateral and inferomedial maxillary wall on right side (arrow) with minimal soft tissue thickening is noted in right orbit abutting inferior rectus muscle (dashed arrow) with a blocked OMU (asterisk)

The patient underwent high-resolution computed tomography (HRCT) scan of thorax at the time of admission and the findings were consistent with a typical COVID-19 infection (Fig. 3). A CT scan of head was advised on the 3rd day after discharge (17th day from the positive RTPCR) when the patient was readmitted to the hospital for the grayish discharge. The CT head revealed a subtle but critical finding, infiltration of the right retro antral fat plane (Fig. 4) which raised the suspicion of it being something other than just a case of simple sinusitis. Complete opacification of the right maxillary sinus and anterior ethmoidal air cells with internal foci of air without an air fluid level was appreciated. Right ostiomeatal unit (OMU) was blocked (Fig. 5). A small defect was noted in posterior-lateral and inferomedial maxillary wall on right side. Minimal soft tissue thickening was noted in right orbit abutting inferior rectus muscle.

**Fig. 6:**
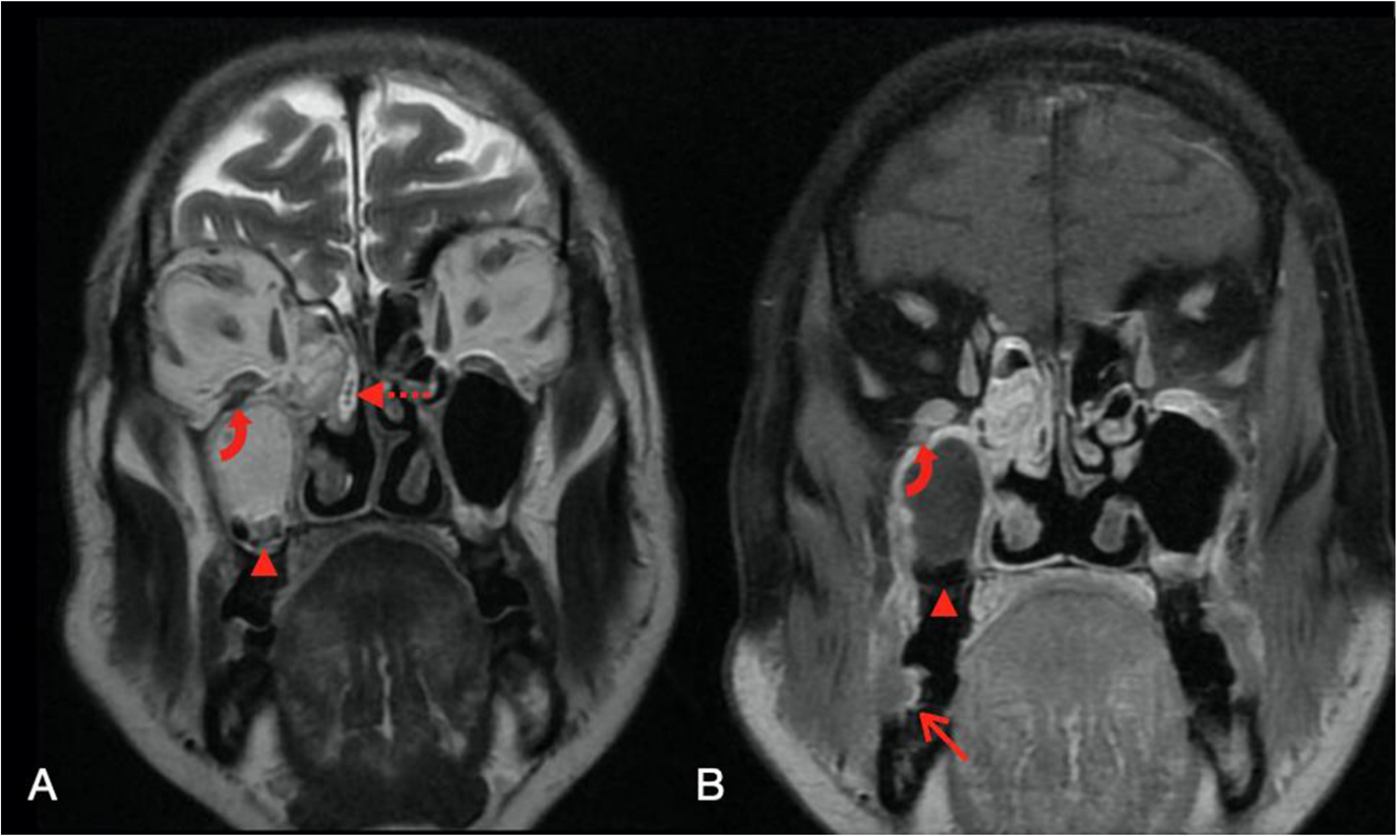
Coronal T2 (**A**) and T1 FS +Contrast (**B**) demonstrating the enhancing mucosal thickening in right ethmoidal and bilateral maxillary sinuses with non-enhancing collection in right maxillary sinus (arrowhead). Enhancing soft tissue extension is noted in right orbit abutting inferior rectus muscle (curved arrow) and extending into right nasolacrimal duct (dashed arrow). Laterally, it is extending to involve right buccinator muscle (thin arrow)

**Fig. 7:**
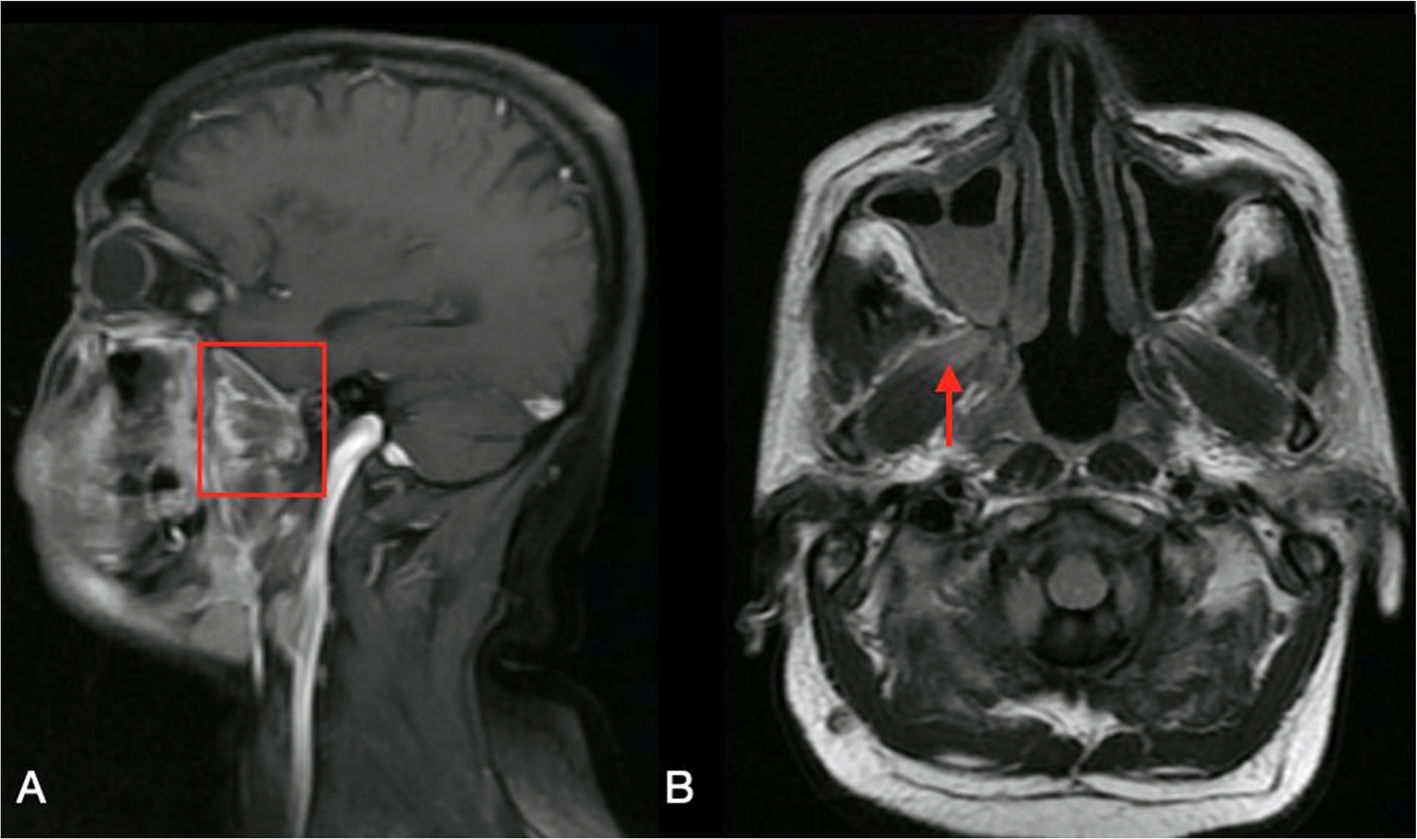
T1 sagittal FS + contrast (**A**) and axial FLAIR (**B**) study there is enhancement of right medial and lateral pterygoid muscles (red box) and subcutaneous soft tissue component anterior to maxilla on right side. Inflammation and involvement of the right pterygoid muscles (red arrow) and parapharyngeal space below it

Two days later, MRI brain (Fig. 6) was performed and showed T1 hypointense and T2 isointense enhancing soft tissue thickening predominantly in the right maxillary and ethmoid sinus extending to involve right pterygoid (Fig. 7) and buccinator muscle, right nasopharynx and pharyngeal mucosal space on right side with intraorbital extension and without any obvious intracranial extension. A small focus of with non-enhancing collection was noted in the right maxillary sinus.

## Discussion

While considering the diagnosis of CAROM, we encountered very few reported cases which had described the radiological aspect of the disease [6–8]. It is postulated that the SARS-CoV-2 infection may affect CD4+ and CD8+ T cells, with a reduction in the absolute number of lymphocytes and T cells associated with creation of a temporary state of compromised immunity. Orbital involvement results from the spread through the medial orbital wall and nasolacrimal duct as in our case. The fungi invade the adjacent blood vessels causing thrombosis and infarction, as well as dissemination to the brain parenchyma. Fortunately, our patient was diagnosed early and progression to the cerebral form of the disease was prevented.

The more aggressive form of the disease with an early cerebral involvement has been depicted well in a few studies [9] where in retro antral, facial, and orbital fat stranding are the initial indicators of the aggressive course [10, 11].

An effective screening tool (Table 1) was used to screen our patient and it is based on the findings on a non-contrast CT and has shown to predict acute invasive fungal sinusitis with 100% specificity [12]. Our case demonstrated four of the seven features. Much like in our case, the early disease manifestation on CT scan is of mucosal thickening usually without air fluid levels [13].The MRI of the sinuses and orbits in ROM is documented to show three patterns [14] with a majority of the cases showing iso- to hypointense appearance on T2, we appreciated an isointense pattern in our case. The T2 hypointense to isointense appearance may be due to presence of iron and manganese in the fungal elements [15]. As described in previous literature, MRI has proved to be very useful in detection of complications like orbital cellulitis, cavernous sinus thrombosis, and ICA thrombosis [16].

**Table 1:** The 7 variable CT-based model [12]

Parameters on non-contrast CT	Acute invasive fungal sinusitis
o Involvement of	
• Pterygopalatine fossa	
• Nasolacrimal duct	Considered positive if any 2 of the 7 features are present
• Lacrimal sac	
o Periantral fat stranding	
o Bony dehiscence	
o Nasal septal ulceration	
o Orbital invasion	

Another important radiological finding that has been widely mentioned in literature as relatively specific for the disease is the “Black turbinate sign” [17]. It was from a case report published in 2010 on the basis of two cases of rhino cerebral mucormycosis and it highlighted the MR imaging findings associated with devitalization of the sinonasal mucosa caused by mycotic vascular invasion. Which meant parts of the mucosa would show non enhancement on contrast. Intriguingly, none of the reported CAROM cases characterize the similar appearance or mention it. As in our case, there was no apparent focal devitalization or ischemia of the mucosa which coerces us to think if the sign is really specific for CAROM.

## Conclusion

The importance of keeping an eye out for the subtle but critical early imaging findings as described in a known COVID-19 case with symptoms of sinusitis is imperative. Culture and microbiological confirmation takes time usually a few days by using the 7 variable CT model [13] as used in our case can be of help while awaiting the laboratory diagnosis. The typical signs such as the “Black turbinate sign” [17] may not always be seen in the early stages of the disease and suspected individuals should not be dismissed based on the absence of radiologically visible ischemic sinonasal mucosa.

## Data Availability

Not applicable

## Abbreviations

CAROM: COVID-19-associated rhino-orbital mucormycosis
CAM: COVID-19-associated mucormycosis
ROM: Rhino-orbital mucormycosis
RT-PCR: Reverse transcription polymerase chain reaction
ICU: Intensive care unit
HRCT: High-resolution computed tomography
MRI: Magnetic resonance imaging
CT: Computed tomography
OMU: Ostiomeatal unit
ICA: Internal carotid artery
